# THE BRAZILIAN ARCHIVES OF DIGESTIVE SURGERY COMPLETES 35 YEARS AND A NEW CYCLE BEGINS

**DOI:** 10.1590/0102-672020210002e1623

**Published:** 2022-01-31

**Authors:** Nelson Adami ANDREOLLO, Francisco TUSTUMI, José Eduardo de AGUILAR-NASCIMENTO

**Affiliations:** 1 Professor Titular de Cirurgia, Departamento de Cirurgia, Faculdade de Ciências Médicas da Universidade Estadual de Campinas - Unicamp, Campinas - São Paulo, Brasil; 2 Departamento de Gastroenterologia, Hospital de Clínicas da Faculdade de Medicina da Universidade de São Paulo - HCFMUSP, São Paulo - São Paulo, Brasil; 3 Professor Titular, Departamento de Clínica Cirúrgica, Faculdade de Medicina da Universidade Federal de Mato Grosso (UFMT) - Cuiabá, Mato Grosso, Brasil. Diretor do Curso de Medicina do Centro Universitário de Várzea Grande (UNIVAG)

**Figure 1 -:**
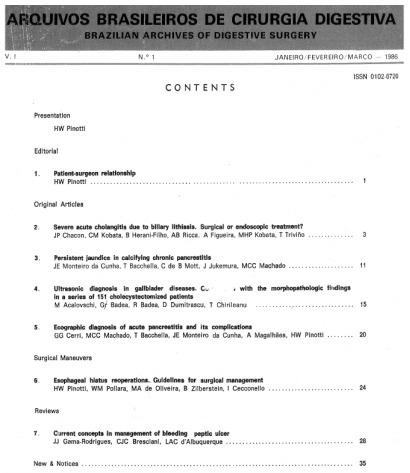
Articles that formed number 1, volume 1, January-March 1986.

The *Brazilian Archives of Digestive Surgery* (*ABCD*) in 2021 completes 35 years of uninterrupted publications. It was conceived by Professor Henrique Valter Pinotti, its founder and first editor, in 1986 ^1^ and had its first issue published at the beginning of the same year (Figure 1). All these years, the *ABCD*- as well described in the words of Professor Osvaldo Malafaia ^4^, its editor for 20 years (2001-2021)-followed a victorious trajectory!

The *ABCD* has published scientific papers produced by Brazilian and foreign authors and researchers, contributing to the dissemination of advances in digestive surgery, gastroenterology, digestive endoscopy, and nutrition. Over the past 35 years, approximately 1650 scientific papers were published ^5^.

In recent years, the *ABCD* has received submissions of national and scientific papers from authors from several other countries, such as the United States, England, Spain, France, Belgium, Germany, Mexico, Chile, Argentina, Peru, Cuba, India, Saudi Arabia, Turkey, China, Iran, and others, demonstrating its gain in prestige and international reputation ^5^.

There were years of enormous efforts and continuous dedication of its editors, associate editors, and members of the editorial board, analyzing scientific works and guiding their authors so that the best of digestive surgery in the country and the world could be published and disseminated.

Professor Henrique Valter Pinotti, with his futuristic perception of an educator, researcher, and excellent surgeon, was also the creator of the *Brazilian College of Digestive Surgery* (*CBCD*), founded in 1988. The *CBCD*, since then, with the mission of to congregate digestive surgeons, through its presidents and boards, has been dedicated to the scientific improvement of its members, in defense of professional practice and working conditions, in order to offer safety and quality to the professional in the performance of their function ^8^.

In the early 1990s, Professor Henrique Valter Pinotti integrated the *ABCD* with *CBCD*, aiming to reach young surgeons and residents of digestive surgery as well and thus contributing to their training, helping them treat the most severe and complex patients, as well as successfully performing large-scale surgical interventions. In addition, he aimed to disseminate research from postgraduate courses ^5^ ^,^ ^6^ ^,^ ^7^.

The *ABCD* editors, in chronological order, were as follows:

- 1986-1990-editorial board-Professors Angelita Habr-Gama, Bruno Zilberstein, Ivan Cecconello, Joaquim Gama-Rodrigues, and Marcel Cerqueira Cesar Machado
- 1991-2000-Professor Bruno Zilberstein
- 2001-2021-Professor Osvaldo Malafaia

In 2021, a new editorial board is challenged to maintain the quality and expand the prestige of the periodic. The new editorial board is being reformulated and will include national and foreign specialist surgeons, from specific areas, such as digestive surgery (benign and oncological diseases), obesity and metabolic surgery, liver surgery, biliary and pancreas surgery, videosurgery and robotics, coloproctology, organ transplantation, nutrition, and perioperative care.

Professor Osvaldo Malafaia, without a doubt, was the editor who contributed for the periodic to reach the current level of visibility, indexing it to SciELO (Scientific Electronic Library Online) (since 2007), to Medline/PubMed (since 2012), to PubMed Central (since 2014) and other worldwide indexing bases (Scopus/SCImago, Web of Science - Emerging Sources Citation Index [ESCI], Google Scholar, LILACS, and DOAJ) ^3^ ^,^ ^4^ ^,^ ^5^ ^,^ ^6^.

Thus, the current board of *CBCD* recognized and appointed Professor Osvaldo Malafaia as **EDITOR EMERITUS** of the *ABCD*.

All presidents and their respective boards of directors of the *CBCD*, directly and indirectly, contributed and believed in the necessary investments, for the success of the *ABCD*. The presidents of the *CBCD* (1988-2021) were as follows:

- Professor Henrique Walter Pinotti (1988/1990), founder and first president (University of São Paulo - USP, São Paulo, SP)
- Professor Luiz Sérgio Leonardi (1991/1992) (State University of Campinas - UNICAMP, Campinas, SP)
- Professor Edmundo Machado Ferraz (1993/1994) (Federal University of Pernambuco - UFPE, Recife, PE)
- Professor Luiz Rohde (1995/1996) (Federal University of Rio Grande do Sul - UFRS, Porto Alegre, RS)
- Professor Alcino Lazaro Silva (1997/1998) (Federal University of Minas Gerais - UFMG, Belo Horizonte, MG)
- Professor Osvaldo Malafaia (1999/2000) (Federal University of Paraná - UFPR, Curitiba, PR)
- Professor Joaquim José Gama-Rodrigues (2001/2002) (University of São Paulo - USP, São Paulo, SP)
- Professor Paulo Roberto Rocha Savassi (2003/2004) (Federal University of Minas Gerais - UFMG, Belo Horizonte, MG)
- Professor Julio Cesar Uilli Coelho (2005/2006) (Federal University of Paraná - UFPR, Curitiba, PR)
- Professor Angelita Habr-Gama (2007/2008) (University of São Paulo - USP, São Paulo, SP)
- Professor Nelson Adami Andreollo (2009/2010) (State University of Campinas - UNICAMP, Campinas, SP)
- Professor Cleber Dario Pinto Kruel (2011/2012) (Federal University of Rio Grande do Sul - UFRS, Porto Alegre, RS)
- Professor Ivan Cecconello (2013/2014) (University of São Paulo - USP, São Paulo, SP)
- Professor Bruno Zilberstein (2015/2016) (University of São Paulo - USP, São Paulo, SP)
- Professor Nicolau Gregori Czeczko (2017/2018) (Federal University of Paraná - UFPR, Curitiba, PR)
- Professor Delta Madureira Filho (2019/2020) (Federal University of Rio de Janeiro, Rio de Janeiro, RJ)
- Professor Luiz Augusto Carneiro D’Albuquerque (2021/2022) (University of São Paulo - USP, São Paulo, SP)
- Professor Antonio Carlos Ligocki Campos (2023-2024; elected) (Federal University of Paraná - UFPR, Curitiba, PR)

The current editors of the *ABCD* will maintain the guidelines of previous editors in order to promote digestive surgery, in addition to maintaining and expanding the universe of achievements gathered by Professor Osvaldo Malafaia.

The *ABCD* will continue to publicize progress in the etiopathogenesis and diagnosis of diseases, experimental research related to the digestive system, perioperative care, traditional surgery, new surgical techniques, experimental surgery, systematic reviews and meta-analyses, videosurgery, bariatric and metabolic surgery, oncological surgery in the digestive tract, nutrition in surgery, endoscopic surgery, and, more recently, the robotic surgery.

Over the past 35 years, the *ABCD* has circulated in printed form among surgeons and members of the *CBCD*. After 2007, it began to be published fully bilingually and also online, with free access for obtaining all published articles, without costs, via SciELO, Medline/PubMed, and PubMed Central, in all editions. With the current facilitated access to indexing databases around the world and wide access to tablets, cell phones, notebooks, and microcomputers, from 2022, it will be published only online, in the indexing databases. Furthermore, to expand the journal’s internationalization, all publications will be in English.

Considering the growing interest of the nonspecialist public in scientific topics and the need for social contribution, the current editorial board of the *ABCD* proposes to approach the general public, without losing, however, attention to its specialist public. Network communication technologies grow day by day, which can be an efficient tool to promote the circulation of information.

Thus, from 2022, the *ABCD* proposes to insert itself in the so-called social networks: Facebook, Twitter, YouTube, Instagram, Wikipedia, and blogs. Social networks are popular platforms, which open up the possibility of expanding the distribution of news, not only of interest to specialist physicians but also of interest to the nonspecialist public ^2^. So, in this way, the *ABCD* aims to contribute to the teaching and dissemination of research and evidence-based medicine.

New national and international reviewers will participate in the peer-review process of each article submitted to the journal. The body of reviewers will cover all areas of knowledge in the digestive system, in its various sub-specialties, so that each manuscript is well evaluated and that the reviews contribute significantly to the improvement of the article, before its publication. In this scenario, the participation of international reviewers will be crucial for the broad exchange of scientific knowledge.

Looking to facilitate the submission process and the expansion of the *ABCD*, the journal’s electronic address will be redesigned for 2022. The new website will bring resources to favor the submission of new manuscripts by authors so that access to the journal’s content is widely disseminated.

In this way, the intention of the editors is to consolidate the achievements obtained over the past 35 years of the *ABCD* and to expand the dissemination of science internationally and in the most diverse audiences, specialists, and nonspecialists. Thus, the *ABCD* will extend its social importance and contribute to the advancement and propagation of research in digestive surgery, in Brazil and worldwide, and consequently will gain more impact, prestige, and reputation.
